# Genetic variations in extracellular matrix degradation pathways linking to major depressive disorder: Evidence from a large-scale genetic association study

**DOI:** 10.37796/2211-8039.1677

**Published:** 2025-12-01

**Authors:** Halliru Zailani, Daniel Tzu-Li Chen, Sheng-Che Lin, Jia-Hau Lee, Mei-Ling Li, Jane Pei-Chen Chang, Kuan-Pin Su

**Affiliations:** a Mind-Body Interface Research Center (MBI-Lab), China Medical University Hospital, Taichung, Taiwan; b Graduate Institute of Nutrition, China Medical University, Taichung, Taiwan; c Department of Chinese Medicine, China Medical University Hospital, Taichung, Taiwan; d An-Nan Hospital, China Medical University, Tainan, Taiwan; e Child and Adolescent Psychiatry Division, Department of Psychiatry, China Medical University Hospital, Taichung, Taiwan; f Office of Research and Development, Asia University, Taichung, Taiwan; g College of Medicine, China Medical University, Taichung, Taiwan

**Keywords:** Blood-brain barrier, Extracellular matrix, Genome-wide association study, Major depressive disorder, Single nucleotide polymorphisms

## Abstract

Dysregulation of extracellular matrix (ECM) degradation pathways has been increasingly implicated in major depressive disorder (MDD), yet its genetic basis remains unclear. This study investigated the relationship between ECM-related genetic polymorphisms and MDD susceptibility. In a case-control study, we analyzed 317 MDD patients and 1268 sexmatched controls from the Taiwan Biobank (TWB). Genomic DNA was analyzed using the Affymetrix TWB array, targeting single nucleotide polymorphisms (SNPs) in 140 ECM degradation genes (Reactome database). Full-model association tests identified significant SNPs, validated with 5000 max(T) permutations and adjusted via logistic regression for age, body mass index, education level, and marital status. We identified 12 SNPs across 10 ECMrelated genes significantly associated with MDD, including ADAM metallopeptidase domain 17 (*ADAM17*, rs55820761), brevican (*BCAN*, rs11264511), CD44 molecule (*CD44*, rs12270356), collagen type XVII alpha 1 chain (*COL17A1*; rs2282436 and rs10883962), collagen type III alpha 1 chain (*COL3A1*; rs16830979 and rs10883962), collagen type VI alpha 6 chain (*COL6A6*, rs16830219), cathepsin L (*CTSL*, rs2274611), Kallikrein-related peptidase 2 (*KLK2*, rs2664156), matrix metallopeptidase 11 (*MMP11*, rs738791), and nicastrin (*NCSTN*, rs3753391). This study provides the first genetic evidence linking ECM degradation pathways to MDD susceptibility, identifying novel biomarkers for early diagnosis and precision therapy. Further research and cross-population studies are needed to confirm these findings.

## 1. Introduction

Major depressive disorder (MDD) is a highly prevalent and debilitating psychiatric disorder that significantly contributes to global disability and healthcare burden [1,2]. While previous research has primarily focused on neurotransmitter imbalances and neuroinflammation, emerging evidence suggests that extracellular matrix (ECM) remodeling also plays a crucial role in its pathophysiology [3–5]. The ECM, a dynamic structural network composed of proteoglycans, glycoproteins, and fibrous proteins such as collagen and laminins, is critical for maintaining synaptic plasticity and neuronal stability, and blood-brain barrier (BBB) integrity [6]. Disruptions in ECM homeostasis, particularly excessive ECM degradation, have been implicated in synaptic dysfunction, neuronal vulnerability, and neurovascular instability [7,8], which are hallmark pathological features observed in MDD. ECM degradation, particularly via matrix metalloproteinases (MMPs), disrupts BBB integrity in MDD, allowing inflammatory mediators to exacerbate neuroinflammation and neuronal damage [9].

Several families of collagenases and enzymes involved in ECM remodeling, including MMPs, cathepsins, plasminogen activators (PAs), and disintegrin and metalloproteinase with thrombospondin motifs (ADAMTS) proteins, have been implicated in neuropsychiatric disorders [9–13]. MMPs, particularly MMP-9 and MMP-2, play a central role in ECM degradation, affecting synaptic remodeling, neuroinflammation, and BBB permeability [14,15]. Cathepsins, lysosomal enzymes involved in ECM turnover, have been linked to neuronal apoptosis and synaptic dysfunction [16]. Plasminogen activators (tPA and uPA) facilitate ECM degradation by activating MMPs, amplifying ECM remodeling [17]. The ADAMTS protein family regulates ECM glycoprotein turnover, including aggrecan and versican, which are essential for synaptic stability [18,19]. On the other hand, several members of the collagen superfamily play essential roles in neuronal maturation, circuit formation, axon guidance, synaptogenesis, and the maintenance of BBB [13]. Indeed, dysregulation of these proteins and enzymes may aggravate inflammation, compromise neurovascular integrity, and impair synaptic plasticity, potentially contributing to MDD.

Furthermore, this dysregulation can result in the structural and functional impairment of the BBB, which is increasingly recognized as a key factor in MDD pathogenesis, as compromised integrity allows peripheral immune cells and inflammatory mediators to enter the brain, exacerbating neuroinflammation and neuronal damage [9]. Elevated activity of ECM degrading enzymes, particularly MMPs and plasminogen activators, has been linked to increased BBB permeability [20,21], reinforcing the role of ECM remodeling in MDD-related neurovascular dysfunction. Importantly, genetic polymorphisms affecting ECM-degrading enzymes may predispose individuals to BBB instability, increasing susceptibility to MDD. However, human genetic studies exploring ECM-related gene variants and their association with MDD remain scarce, hence, research is needed to uncover the genetic underpinnings of ECM dysregulation in MDD.

In this study, we conducted a case-control genome-wide association study to examine whether polymorphisms in ECM-related genes affect MDD susceptibility. We hypothesized that genetic variations in ECM-degrading enzymes influence MDD risk. Our findings could provide novel insights into the role of ECM degradation and MDD, highlighting potential biomarkers for early diagnosis and personalized treatment. Identifying genetic risk factors associated with ECM dysregulation could facilitate precision medicine approaches, enhancing therapeutic strategies for MDD. Moreover, understanding ECM’s role in depression may reveal common molecular pathways shared with other neuropsychiatric and neurovascular disorders, expanding the implications for psychiatric genetics and neurobiology.

## 2. Methods

### 2.1. Study design and participants

The study was a case-control study involving patients with MDD and healthy controls. The methods used in this study have been described previously [22]. Briefly, adult patients diagnosed with MDD were recruited based on the diagnostic criteria outlined in the *Diagnostic and Statistical Manual of Mental Disorders, Fifth Edition (DSM-5)*. Eligibility required a Hamilton Depression Rating Scale (HAM-D) score of >18, indicating moderate to severe depression. Patients with comorbid psychiatric disorders (e.g., bipolar disorder, schizophrenia), active suicidal ideation, or substance use disorders were excluded. A total of 327 patients meeting these criteria were enrolled as the case group.

The control group consisted of sex-matched healthy individuals selected from the Taiwan Biobank (TWB) at a 4:1 ratio relative to the case group. To minimize potential undiagnosed MDD cases, only participants aged ≥65 years were included, as this demographic is less likely to develop de novo MDD, significantly reducing the likelihood of including individuals with undiagnosed or subsyndromal MDD in the control group. Furthermore, having older individuals in the control group reduces the likelihood of including cases of late-onset depression, resulting in a more robust and well-defined control population, which is particularly important for enhancing the validity of genetic association studies. All participants provided written informed consent, received educational materials on MDD, and were offered access to treatment options, including antidepressants and psychosocial interventions, in accordance with the Institutional Review Board-approved protocols.

### 2.2. Candidate gene selection

Candidate genes were identified using Reactome database pathway annotations (https://reactome.org, accessed on Jan 14, 2025). Specifically, the entry “Degradation of the extracellular matrix” (R-HSA-1474228) was used to identify genes involved in the ECM degradation pathways. A total of 140 genes were identified and included in our subsequent analysis.

### 2.3. Genotyping

Genomic DNA was extracted from buffy coat samples using the QIAamp DNA Blood Mini Kit (Qiagen, Crawley, UK). Genotyping was performed using the Affymetrix TWB array, a genome-wide genotyping platform developed by Thermo Fisher Scientific (MA, USA). This high-density array encompasses >600,000 single nucleotide polymorphisms (SNPs), incorporating data from major international reference panels such as the HapMap Project and the 1000 Genomes Project. The array was designed for ethnic-specific genetic adaptation, ensuring broad applicability across diverse populations. Genotyping was conducted at the National Center for Genome Medicine, Academia Sinica, Taipei, Taiwan.

### 2.4. Data quality control

Quality control (QC) procedures were performed using Plink software (v1.90b6.24) to ensure the accuracy and reliability of genomic data. For participant QC, sex verification was conducted to confirm consistency between reported and chromosomal sex, and clinical data were validated to ensure accurate MDD diagnoses and medical histories. Participants with discrepancies between assigned and chromosomal sex or those with ambiguous clinical records were excluded, however, no additional exclusions were made. For SNP QC, data retention criteria included a genotyping call rate ≥95 %, a minor allele frequency (MAF) >0.01, and compliance with Hardy–Weinberg equilibrium (HWE) at p > 0.001 in control subjects [23]. After QC filtering, genotyping success rates were 99.7 % for MDD cases and 98.7 % for controls. A total of 317 MDD cases and 1268 healthy controls met the QC thresholds and were included in the final genetic association analyses.

### 2.5. Statistical analysis

Statistical analyses were conducted using Plink v1.90b6.24 to examine the association between MDD and candidate gene variants. Case-control association analyses were performed using allelic, dominant, and recessive genetic models to identify significant single nucleotide polymorphisms (SNPs) within the candidate genes. To control for genome-wide family-wise error rate (FWER), a 5000-permutation max(T) test was applied, generating empirical p-values by comparing the best original result of each significant SNP against all others. This genome-wide permutation approach, powered by an accelerated expectationmaximization algorithm, has been widely recognized as a robust method for evaluating the combined effects of multiple genetic variants and accounting for linkage disequilibrium (LD). SNPs with empirical p-values of <0.05 were considered genome-wide significant in accordance with previous studies [22,24,25]. Additionally, logistic regression models were employed to evaluate the predictive value of each significant SNP. To enhance model accuracy, demographic and clinical covariates, including, age, marital status, education level, and body mass index (BMI), were incorporated into the logistic regression framework. Functional predictions of the significant SNPs were also conducted using SNiPA [26] and RegulomeDB [27].

## 3. Results

### 3.1. Demographic and clinical characteristics of the MDD patients and healthy controls

Table 1 presents the demographic and clinical characteristics of 317 patients with MDD and 1268 healthy controls. MDD patients were significantly younger and had lower BMI compared to controls (all p < 0.001). Education levels and marital status differed significantly between the MDD patients and healthy controls.

### 3.2. Information about the genes with significant SNPs

Out of the 140 ECM-related genes identified through the Reactome database, we found 10 genes with at least 1 SNP significantly associated with MDD. These genes included ADAM metallopeptidase domain 17 (*ADAM17)*, brevican (*BCAN*), CD44 molecule (*CD44*), collagen type XVII alpha 1 chain (*COL17A1*), collagen type III alpha 1 chain (*COL3A1*), collagen type VI alpha 6 chain (*COL6A6*), cathepsin L (*CTSL*), Kallikrein-related peptidase 2 (*KLK2*), matrix metallopeptidase 11 (*MMP11)* and nicastrin (*NCSTN* ) genes (Table 2).

### 3.3. Full-model case-control association test

The full-model association test, which included allelic, dominant, and recessive genetic models, was conducted to identify SNPs associated with MDD. SNPs within the candidate genes in the ECM degradation pathways were considered significant if they had an empirical p-value <0.05, as determined by 5000 genome-wide max(T) permutations. Notably, we identified 12 SNPs significantly associated with MDD under the dominant genetic model (Table 3). These SNPs included: rs55820761 in *ADAM17* ( *p* = 0.03, minor allele frequency [MAF] in cases vs. controls = 0.041 vs. 0.0675), rs11264511 in *BCAN* ( *p* = 0.04, MAF = 0.317 vs. 0.3603), rs12270356 in *CD44* ( *p* < 0.001, MAF = 0.1577 vs. 0.1113), two SNPs in *COL17A1* (rs2282436 (( *p* = 0.04, MAF = 0.1772 vs. 0.1337) and rs10883962 ( *p* = 0.03, MAF = 0.1767 vs. 0.1313)), two SNPs in *COL3A1* (rs16830979 ( *p* = 0.002, MAF = 0.1614 vs. 0.1101) and rs12105340 ( *p* = 0.013, MAF = 0.068 vs. 0.1089)), rs16830219 in COL6A6 ( *p* = 0.006, MAF = 0.0852 vs. 0.1418), rs2274611 in *CTSL* ( *p* = 0.03, MAF = 0.3722 vs. 0.3266), rs2664156 in *KLK2* ( *p* = 0.033, MAF = 0.2098 vs. 0.1654), rs738791 in *MMP11* ( *p* = 0.004, MAF = 0.3675 vs. 0.3092), and rs3753391 in *NCSTN* (rs3753391, *p* = 0.006, MAF = 0.0568 vs. 0.0916). All significant SNPs were located in the intronic regions, suggesting that they do not directly alter protein structures (Supplementary Table 1).

### 3.4. Logistic regression

The dominant genetic model was selected based on both our findings and previous recommendations [28]. Logistic regression analysis revealed significant associations between MDD and all 12 identified SNPs. The most robust associations were observed for rs16830979 in *COL3A1* ( *p* < 0.001, odds ratio [OR] = 1.56) and rs16830219 in *COL6A6* ( *p* < 0.001, OR = 0.56), suggesting a strong genetic contribution of these SNPs to ECM degradation pathways in the pathophysiology of MDD (Table 4).

Additional SNPs demonstrating significant associations in the logistic regression included rs55820761 in *ADAM17* ( *p* = 0.01, OR = 0.59), rs11264511 in *BCAN* ( *p* = 0.04, OR = 0.82), rs12270356 in *CD44* ( *p* = 0.001, OR = 1.5), rs2282436 in *COL17A1* ( *p* = 0.005, OR = 1.39), rs2274611 in *CTSL* ( *p* = 0.029, OR = 1.22), rs2664156 in *KLK2* ( *p* = 0.008, OR = 1.34), and rs738791 in *MMP11* ( *p* = 0.005, OR = 1.29). Moreover, three significant SNPs were identified in *NCSTN* (rs3753391 ( *p* = 0.005, OR = 0.59), rs12105340 ( *p* = 0.002, OR = 0.59), and rs10883962 ( *p* = 0.003, OR = 1.42)). Adjustments for demographic and clinical covariates, including age, marital status, education level, and BMI, did not significantly alter the associations observed for any of the SNPs.

## 4. Discussion

This study is the first to systematically link ECM degradation gene polymorphisms to MDD susceptibility in a case-control study. Analyzing 317 MDD patients and 1268 controls, we identified 12 significant SNPs across 10 ECM-related genes, highlighting ECM dysregulation as a genetic risk factor for MDD. Protective variants in ADAM17 (rs55820761), BCAN (rs11264511), COL3A1 (rs12105340), COL6A6 (rs16830219), and NCSTN (rs3753391) suggest resilience mechanisms, while risk variants in CD44 (rs12270356), COL17A1 (rs2282436 and rs10883962), COL3A1 (rs16830979), CTSL (rs2274611), KLK2 (rs2664156), MMP11 (rs738791), and NCSTN (rs10883962) indicate vulnerability pathways.

Our first main finding is that protective SNPs in *ADAM17* (rs55820761), *BCAN* (rs11264511), *COL6A6* (rs16830219), *and NCSTN* (rs3753391 and rs12105340) may enhance ECM stability and BBB function. *ADAM17* encodes a metalloprotease involved in pro-inflammatory cytokine processing and synaptic remodeling, which may contribute to neuroprotection by regulating tumor necrosis factor-alpha (TNF-α) signaling and other pro-inflammatory cytokines [29]. Dysregulated TNF-α and other proinflammatory cytokine activity has been linked to MDD [30–33]. Similarly, BCAN is a brain-specific chondroitin sulfate proteoglycan involved in the regulation of synaptic plasticity by inhibiting neurite outgrowth and maintaining the stability of neuronal connections [34], which may promote synaptic stability and neural plasticity that are impaired in MDD [35]. *COL6A6*, which encodes a component of collagen type VI, plays a crucial role in maintaining BBB integrity and providing neuronal protection [13]. Abnormalities in *COL6A6* have been linked to behavioral abnormalities and cortical dopaminergic dysfunction [36] while also promoting neurodegeneration by impairing autophagy and inducing apoptosis [37]. *NCSTN*, a component of the γ-secretase complex, may influence Notch signaling, a pathway essential for neuronal differentiation and synaptic homeostasis [38,39]. Given the role of Notch signaling in neurodevelopmental and mood disorders [40,41], these variants may confer resilience to MDD by preserving synaptic structure and function and improving neurogenesis [42,43]. Notably, all the significant SNPs identified in our study were located in the intronic regions, suggesting that they do not directly alter protein structures. However, these intronic variants may influence gene expression, splicing efficiency, or regulatory mechanisms.

Conversely, we found that SNPs in *CD44* (rs12270356), *COL17A* (rs2282436 and rs10883962), *COL3A1* (rs16830979), *CTSL* (rs2274611), *KLK2* (rs2664156), and *MMP11* (rs738791), were associated with a heightened risk of developing MDD, potentially by disrupting ECM remodeling, synaptic function, and BBB stability. *CD44* encodes a cell surface glycoprotein involved in ECM interactions and inflammation [44,45]. Increased *CD44* expression has been reported in neuroinflammatory conditions [46,47], and its dysregulation may exacerbate microglial activation and glutamate excitotoxicity, both of which have been implicated in MDD [48–50]. Similarly, *COL17A1* and *COL3A1* are essential for neuronal adhesion, ECM stability, vascular integrity, and cerebrovascular dysfunction [51]. Dysfunctional collagen synthesis may impair BBB integrity and function, neurovascular coupling, and promote neuroinflammation and oxidative stress [52,53]—hallmarks of MDD pathogenesis. Other ECM-related genes linked to increased MDD risk include *CTSL, KLK2*, and *MMP11*. CTSL is a lysosomal cysteine protease involved in ECM degradation, synaptic remodeling, and promotion of axonal growth [54,55]. Dysregulation of cathepsins has been implicated in neuroinflammation and cognitive decline [10,56]. KLK2 is a serine protease known to regulate ECM turnover and inflammation, which may contribute to MDD by affecting synaptic remodeling and microglial activation [57]. MMP11, like other MMP family members, plays a role in ECM degradation and neurovascular remodeling [58]; aberrant MMP activity has been linked to increased BBB permeability and neuroinflammation [59], both of which have been implicated in MDD [60,61].

Our findings have important clinical implications, particularly in advancing precision medicine for MDD. The identification of genetic risk factors associated with ECM degradation and BBB dysfunction could lead to personalized treatment approaches, including the development of targeted therapeutics aimed at stabilizing ECM integrity and improving BBB function. For instance, MMP inhibitors, which have been explored in neurological and inflammatory diseases, could be repurposed to mitigate ECM-driven BBB dysfunction in MDD. Additionally, genetic screening for the identified SNPs may aid in the early identification of individuals at higher risk for MDD, enabling preventive interventions such as anti-inflammatory or neuroprotective strategies. Future research should explore whether MDD patients with these specific ECM-related SNPs exhibit differential responses to antidepressant treatment, particularly in relation to anti-inflammatory agents or neurotrophic interventions. Notably, omega-3 polyunsaturated fatty acids (omega-3) have been documented to possess antidepressant effects via anti-inflammatory and neuroprotective mechanisms [62–66], and genetic screening of the identified SNPs in individuals with MDD may aid in the optimization of treatment response to omega-3.

Our study has some limitations. First, our study population was derived from Taiwan, and while this ensures a homogeneous genetic background, it may limit generalizability to other populations. Similarly, formal assessment and adjustment for population stratification using principal component analysis (PCA) were not performed. This raises the possibility of residual confounding due to subtle population structure, which may influence genetic association results. Furthermore, although single-SNP logistic regression analyses adjusted for relevant covariates were conducted, we did not perform multivariate logistic regression incorporating all significant SNPs simultaneously. This limits the ability to assess independent SNP effects, account for potential multicollinearity due to linkage disequilibrium, and evaluate combined genetic risk. While we identified statistically significant SNP associations, further functional studies using in vitro and in vivo models are warranted to elucidate the precise mechanistic roles of these SNPs in MDD pathophysiology. Finally, although we adjusted for potential confounding variables (age, BMI, education, marital status), additional factors such as lifestyle, stress exposure, and environmental influences may also modulate ECM-related MDD risk and warrant further investigation. Thus, future research should focus on replicating these findings and address these limitations by incorporating population stratification adjustment using PCA and multivariate analyses, and exploring gene-environment interactions in diverse and longitudinal cohorts to determine whether these SNPs influence disease progression and treatment response in MDD patients.

## 5. Conclusion

This study firstly provides genetic evidence linking ECM degradation pathways to MDD susceptibility, identifying 12 significant SNPs across 10 ECM-related genes. These findings introduce potential genetic biomarkers for the early diagnosis and personalized treatment of MDD. Future functional studies and replication in diverse populations are needed to confirm these associations and to translate them into clinical practice.

## Supplementary Material

**Supplementary Table 1:** Functional Annotations of the Significant SNPs.

Gene	SNP	Putative effect on transcript	SNP effect impact	CADD Score	Conservation (GERP++)	RegulomeDB Rank
*ADAM17*	rs55820761	Intronic	Modifier	7.394	−2.3	2b
*BCAN*	rs11264511	Intronic	Modifier	0.326	−3.14	1f
*CD44*	rs12270356	Intronic	Modifier	7.539	1.6	4
*COL17A1*	rs2282436	Intronic	Modifier	5.522	−1.35	1f
	rs10883962	Intronic	Modifier	1.139	−5.39	1f
*COL3A1*	rs16830979	Intronic	Modifier	12.62	−3.12	2b
	rs12105340	Intronic	Modifier	0.926	2.29	7
*COL6A6*	rs16830219	Intronic	Modifier	11.99	2.23	1f
*CTSL*	rs2274611	Intronic	Modifier	1.17	−2.69	1f
*KLK2*	rs2664156	Intronic	Modifier	2.967	−1.66	1d
*MMP11*	rs738791	Intronic	Modifier	1.928	−4.68	1b
*NCSTN*	rs3753391	Intronic	Modifier	5.332	−0.596	4

ADAM17: ADAM metallopeptidase domain 17, BCAN: brevican, CADD: combined annotation dependent depletion, CD44: CD44 molecule, CTSL: cathepsin L, COL17A1: collagen type XVII alpha 1 chain, COL3A1: collagen type III alpha 1 chain, COL6A6: collagen type VI alpha 6 chain, GERP++: Genomic evolutionary rate profiling, KLK2: kallikrein-related peptidase 2, MMP11: matrix metallopeptidase 11, NCSTN: nicastrin, SNP: single nucleotide polymorphism.

## Figures and Tables

**Table 1:** Demographic and clinical characteristics of the cases and controls.

Variable	MDD patients (n = 317)	Healthy controls (n = 1268)	P-value
**Age** (years, mean ± SD)	40.89 ± 14.32	66.90 ± 1.64	<0.001
**Sex** (% male)	19.56	19.56	1.000
**BMI** (kg/m^2^)	22.67 ± 3.55	24.18 ± 3.37	<0.001
**Education level** (%)			<0.001
No formal education	1.80	1.80	
Self-taught	0.10	0.50	
Primary	7.62	21.90	
Junior high school	9.81	15.61	
Senior high school	29.10	28.50	
College	44.30	27.61	
Graduate school and above	7.20	4.10	
**Marital status** (%)			<0.001
Single	32.10	2.40	
Married	67.50	71.20	
Divorced/separated	0.10	5.60	
Spouse deceased	0.30	20.80	

Data presented as mean ± SD or percentage. Independent T-test was used to assess differences in age and BMI. The differences in sex, education level, and marital status were assessed using Chi-square. BMI: Body mass index, MDD: Major depressive disorder.

**Table 2:** Information of genes with significant SNPs.

Gene	Product protein	Location	Length
ADAM17	TACE - Tumor Necrosis Factor-Alpha-Converting Enzyme	2	67344
BCAN	Brevican	1	17411
CD44	Cluster of Differentiation 44	11	93231
COL17A1	Collagen Type XVII Alpha 1 Chain	10	54594
COL3A1	Collagen Type III Alpha 1 Chain	2	38373
COL6A6	Collagen Type VI Alpha 6 Chain	3	159865
CTSL	Cathepsin L.	9	5350
KLK2	Kallikrein-related peptidase 2	19	7128
MMP11	Matrix Metalloproteinase-11	22	11467
NCSTN	Nicastrin	1	15566

ADAM17: ADAM metallopeptidase domain 17, BCAN: brevican, CD44: CD44 molecule, CTSL: cathepsin L, COL17A1: collagen type XVII alpha 1 chain, COL3A1: collagen type III alpha 1 chain, COL6A6: collagen type VI alpha 6 chain, KLK2: kallikrein-related peptidase 2, MMP11: matrix metallopeptidase 11, NCSTN: nicastrin. SNP: single nucleotide polymorphism.

**Table 3:** Full-model case-control association analysis of the significant genes.

Genes	SNP	Empirical p-value	Minor Allele	Major Allele	MAF in cases	MAF in controls
*ADAM17*	rs55820761	0.0363	T	C	0.0410	0.0675
*BCAN*	rs11264511	0.0422	A	G	0.3170	0.3603
*CD44*	rs12270356	0.0477	A	G	0.1577	0.1113
*COL17A1*	rs2282436	0.0496	C	A	0.1772	0.1337
	rs10883962	0.0320	C	T	0.1767	0.1313
*COL3A1*	rs16830979	0.0020	T	C	0.1614	0.1101
	rs12105340	0.0126	T	G	0.0680	0.1089
*COL6A6*	rs16830219	0.0067	T	C	0.0852	0.1418
*CTSL*	rs2274611	0.0322	T	C	0.3722	0.3266
*KLK2*	rs2664156	0.0374	T	C	0.2098	0.1654
*MMP11*	rs738791	0.0052	T	C	0.3675	0.3092
*NCSTN*	rs3753391	0.0055	T	C	0.0568	0.0916

ADAM17: ADAM metallopeptidase domain 17, BCAN: brevican, CD44: CD44 molecule, CTSL: cathepsin L, COL17A1:collagen type XVII alpha 1 chain, COL3A1: collagen type III alpha 1 chain, COL6A6: collagen type VI alpha 6 chain, KLK2: kallikrein-related peptidase 2, MMP11: matrix metallopeptidase 11, NCSTN: nicastrin, MAF: Minor allele frequency, SNP: single nucleotide polymorphism. Empirical p-value: the p-value obtained with the permutation technique. p-value means the p-values obtained in the original chi-squared test.

**Table 4:** Logistic regression results of the significant genes.

Gene	SNP	p-value	Odds ratio (95 % CI)
*ADAM17*	rs55820761	0.0136	0.59 (0.23–0.39)
*BCAN*	rs11264511	0.0413	0.82 (0.68–0.99)
*CD44*	rs12270356	0.0013	1.50 (1.16–1.91)
*COL17A1*	rs2282436	0.0050	1.39 (1.11–1.76)
	rs10883962	0.0032	1.42 (1.12–1.79)
*COL3A1*	rs16830979	0.0004	1.56 (1.22–1.99)
	rs12105340	0.0022	0.59 (0.43–0.83)
*COL6A6*	rs16830219	0.0002	0.56 (0.42–0.76)
*CTSL*	rs2274611	0.0296	1.22 (1.02–1.47)
*KLK2*	rs2664156	0.0083	1.34 (1.08–1.67)
*MMP11*	rs738791	0.0049	1.29 (1.08–1.56)
*NCSTN*	rs3753391	0.0049	0.59 (0.42–0.86)

ADAM17: ADAM metallopeptidase domain 17, BCAN: brevican, CD44: CD44 molecule, CTSL: cathepsin L, COL17A1: collagen type XVII alpha 1 chain, COL3A1: collagen type III alpha 1 chain, COL6A6: collagen type VI alpha 6 chain, KLK2: kallikrein-related peptidase 2, MMP11: matrix metallopeptidase 11, NCSTN: nicastrin, MAF: Minor allele frequency, SNP: single nucleotide polymorphism. p-value: the p-values obtained in the original chi-squared test.
